# Safeguarding Children’s Movement Behaviours During Public Health Crises: Lessons From a Global Rapid Scoping Review

**DOI:** 10.1177/11786302261479502

**Published:** 2026-08-14

**Authors:** Leigh M. Vanderloo, Taniya S. Nagpal, Richard Tyler, António L. Palmeira, Emily A. Bonisteel, Brianne A. Bruijns, Alissa J. Burnett, Ankhmaa Byambaa, Valerie Carson, Jordyn M. Cox, Audrey Elford, Rebecca B. Emori, Avril Johnstone, Rachel A. Jones, Sanne L. C. Veldman, Dona S. Yudasmara, Fariba Bannerman, Anthony D. Okely

**Affiliations:** 1 School of Occupational Therapy, 6221 University of Western Ontario, London, Canada; 2 Department of Research & Evaluation, ParticipACTION, Toronto, Canada; 3 Faculty of Kinesiology, Sport, and Recreation, 3158 University of Alberta, Edmonton, Canada; 4 Sport, Physical Activity, Health, and Wellbeing Research Group, Department of Sport and Physical Activity, International Centre for Applied Research with Children, Young People, Pregnant Women and Families, 6249 Edge Hill University, Lancashire, UK; 5 CIDEFES, Universidade Lusófona; CIFI2D, 26706 Universidade Do Porto and International Society of Behavioral Nutrition and Physical Activity, Porto, Portugal; 6 Institute for Physical Activity and Nutrition (IPAN), School of Exercise and Nutrition Sciences, 2104 Deakin University, Burwood, AU-VIC, Australia; 7 Early Start School of Medical, Indigenous and Health Sciences, Faculty of Science, Medicine and Health, 8691 University of Wollongong, Wollongong, AU-NSW, Australia; 8 Population Health and Demography Unit, Papua New Guinea Institute of Medical Research, Eastern Highlands Province, Goroka, Papua New Guinea; 9 General Practice and Primary Care, 3526 School of Health and Wellbeing, University of Glasgow, Glasgow, Scotland; 10 School of Education, 8691 University of Wollongong, Wollongong, AU-NSW, Australia; 11 194970 Department of Public and Occupational Health, Mulier Institute, Utrecht, The Netherlands; 12 Department of Physical Education, Health, and Recreation, 106251 Universitas Negeri Malang (State University of Malang), Malang, Indonesia; 13 Library and Learning Services, 6249 Edge Hill University, Lancashire, UK

**Keywords:** children’s movement behaviours, pandemic preparedness, physical activity, sedentary behaviour, sleep, emergency preparedness, child health, public health policy, COVID-19, health systems resilience

## Abstract

Public health measures implemented during the COVID-19 pandemic were essential for limiting viral transmission but had unintended consequences for children’s movement behaviours, including reduced physical activity, increased sedentary time, and disruptions to sleep. These behaviours are fundamental to healthy physical, mental, and social development and were already suboptimal for many children prior to the pandemic. This commentary draws on findings from a rapid global scoping review of 182 studies conducted across 48 countries between 2020 and 2023 and reflects the consensus position of an international group of movement behaviour researchers. Using qualitative evidence synthesis and narrative analysis, the review examined reported changes in children’s movement behaviours and identified commonly proposed strategies to mitigate harms during public health emergencies. Across diverse geographic and economic contexts, studies consistently described declines in physical activity and increases in sedentary time, alongside variable impacts on sleep. Recommended responses most frequently emphasized education, environmental restructuring, enablement, and policy and service-level actions aimed at supporting healthy movement opportunities during periods of restriction. Collectively, the evidence suggests that children’s movement behaviours were insufficiently considered within emergency response planning and were often treated as secondary outcomes rather than essential determinants of child health. We argue that protecting opportunities for physical activity, active play, and healthy daily movement should be embedded within pandemic recovery and future emergency preparedness efforts. Recognizing movement behaviours as a core component of resilient public health systems may help mitigate unintended harms and better safeguard child health and well-being during future crises.

## Introduction

The COVID-19 pandemic prompted unprecedented public health measures, including school closures, restrictions on organized sport and recreation, and stay-at-home orders. While these measures were effective in reducing viral transmission,^1^ they simultaneously disrupted the social, educational, and environmental systems that support healthy movement behaviours among children. As such, COVID-19 functioned as a large-scale stress test of how well public health systems were equipped to protect children’s physical activity, sedentary behaviour, and sleep during periods of widespread disruption.

Children’s movement behaviours are core components of resilient public health systems. Regular physical activity, limited sedentary time, and adequate sleep support physical health, mental well-being, cognitive development, and social functioning, and they contribute to both immediate and long-term population health outcomes.^2,3^ Importantly, even prior to the pandemic, most children globally were not meeting recommended movement behaviour guidelines.^4^ Pandemic-related restrictions therefore did not create new problems but amplified existing inequities and exposed fragilities in systems that deprioritize movement when crises emerge.

A substantial body of literature has documented changes in children’s movement behaviours during COVID-19, including reductions in physical activity, increases in sedentary time, and disruptions to sleep patterns.^5-7^ However, much of this literature remains descriptive and retrospective, with limited synthesis of how these findings can inform preparedness for future pandemics or other large-scale public health emergencies. COVID-19 revealed that children’s movement behaviours are not primarily failures of individual choice but outcomes of system-level decisions that shape access to movement opportunities. As the global research community moves beyond the acute phase of COVID-19, there is a pressing need to translate this evidence into forward-looking insights that strengthen system resilience and protect children’s health during future disruptions.

Based on evidence from a rapid global scoping review (Supplemental Table 1), this commentary presents a future-oriented perspective from an international group of movement behaviour researchers. Rather than revisiting the impacts of COVID-19 per se, we use the pandemic as a sentinel event to identify actionable lessons for recovery and preparedness. We argue that safeguarding children’s movement behaviours must be an explicit priority in emergency preparedness planning and propose evidence-informed recommendations to future-proof systems, policies, and environments to better support children’s movement behaviours during times of crisis.

### Evidence From a Rapid Global Scoping Review

The position advanced in this commentary is informed by a rapid scoping review of 182 studies examining children’s physical activity, sedentary time, and sleep during the COVID-19 pandemic across 48 countries (Supplemental Table 1). The review identified consistent global patterns, including declines in physical activity and substantial increases in sedentary time among children and youth. Changes in sleep were more variable, with many studies reporting longer sleep duration alongside later bedtimes, delayed wake times, and poorer sleep quality.

Although most studies were conducted in high-income countries, increases in sedentary time were more pronounced in these settings compared with low- and middle-income countries. This pattern may partly reflect differential access to screen-based technologies and remote learning environments, underscoring how structural and socioeconomic contexts shape behavioural responses during public health emergencies.^8^

Using the Behaviour Change Wheel as an organizing framework,^9^ the review synthesized commonly proposed intervention and policy directions (Supplemental Tables 2a and 2b, respectively). These findings support an integrated 24-hour approach in which emergency policies consider their combined effects on physical activity, sedentary behaviour (screen time), and sleep rather than addressing each behaviour in isolation. In practice, this could include incorporating movement breaks and avoiding unnecessarily continuous screen-based instruction during remote learning, providing families with adaptable daily routines, maintaining safe indoor and outdoor activity opportunities, and considering whether measures directed at one behaviour create unintended effects on another. Education, environmental restructuring, and enablement were the most frequently recommended intervention functions, while policy recommendations most often concerned practical guidance, service provision, and environmental and social planning. These approaches should be adapted to local resources, restrictions, and population needs and evaluated for feasibility and equity.

### Implications for Practice

The evidence highlights the importance of preserving opportunities for movement during periods of disruption. Education-based strategies could provide parents, caregivers, and children with brief, age-appropriate, and adaptable suggestions for maintaining physical activity, limiting sedentary time (and screen time), and supporting regular sleep routines within different home environments. Health care providers, educators, and community practitioners can reinforce these messages and connect families with locally available supports. However, individual guidance is unlikely to be sufficient when families lack safe spaces, equipment, time, or access to services. Emergency plans for schools should specify how movement opportunities can be maintained across in-person, hybrid, and remote learning models. During in-person schooling, this may involve retaining scheduled recess and activity breaks and using outdoor spaces or staggered schedules where needed. During hybrid or remote learning, schools could incorporate brief movement breaks into instruction and provide accessible activities requiring little space or equipment, including adaptations for children with disabilities. Implementation should reflect local public health requirements, available resources, and student needs.

### Implications for Policy

Policy recommendations arising from the review emphasize integrated, multisector approaches that embed movement behaviours within emergency preparedness frameworks. Families should receive practical guidance that translates broad movement recommendations into feasible daily actions during periods of confinement.^4^ To be actionable, this guidance should be brief, age-appropriate, accessible, and adaptable to different living environments and available resources. It should offer alternatives when outdoor space, equipment, or organized programming is unavailable and identify relevant supports available through schools, health services, recreation providers, or community organizations. Flexible options are more appropriate than a single prescribed routine.

Without deliberate policy correction, post-pandemic recovery efforts risk entrenching sedentary defaults established during COVID-19, including prolonged screen-based schooling and hybrid learning environments that limit movement opportunities. Guidance should be communicated through aligned core messages across public health authorities, schools, health care providers, and recreation organizations; repeated through trusted channels; adapted for different languages and audiences; and updated transparently as conditions change. Coordinated communication may reduce confusion and reinforce movement as a health priority, but it should complement rather than substitute for accessible opportunities and services.^10^

Continuity of movement-related services should also be incorporated into emergency plans, particularly for children facing structural disadvantages, including those with disabilities, chronic conditions, or limited access to safe play spaces. Depending on local conditions, this may include adapted physical activity and rehabilitation services, school-based supports, community recreation programs, equipment-lending initiatives, accessible remote supports, and managed access to schoolyards, playgrounds, and public parks.^11^ Equitable access requires more than maintaining physical infrastructure and may also require affordable programming, transportation, equipment, disability accommodations, accessible and culturally appropriate communication, and consultation with affected communities.

### Call to Action

The COVID-19 pandemic demonstrated that children’s movement behaviours are highly sensitive to large-scale public health measures. Future emergency responses must explicitly consider how restrictive policies affect children’s daily routines, opportunities for movement, and developmental trajectories. Movement behaviours should be treated as essential health assets rather than discretionary activities during public health emergencies.

We call on policymakers, public health leaders, educators, and practitioners to embed protection of children’s movement behaviours into emergency preparedness planning. This includes establishing ethical and inclusive population-level monitoring of physical activity, sedentary behaviours (and screen use), and sleep before, during, and after emergencies; preserving safe movement space and essential movement-related services; integrating movement into remote learning and service delivery models; and ensuring that guidance for families is clear, feasible, and equitable. Monitoring should be used to identify emerging changes and inequities, guide timely adjustments to policies and services, and evaluate recovery. It should collect only data necessary for a defined public health purpose and include appropriate safeguards for privacy, data security, consent or other appropriate legal and ethical authority, transparent governance, accessibility, and public acceptability. It should not involve unnecessary individual-level tracking.^12^ Embedding these measures in preparedness planning now will help ensure that children’s movement needs are not deprioritized when future public health restrictions are introduced.

## Conclusion

Evidence from this rapid global scoping review, alongside converging findings from other published reviews, demonstrates that children’s movement behaviours are highly sensitive to large-scale public health measures. While some behaviours may recover as restrictions ease, the acute disruptions observed during COVID-19 illustrate how quickly movement opportunities can be constrained when systems are not designed for resilience. Recovery alone is therefore insufficient as a guiding framework.

Future public health emergency responses must be designed with explicit consideration of how restrictive measures affect children’s daily routines, access to movement opportunities, and long-term development. Embedding protections of physical activity, sedentary behaviour, and sleep within emergency preparedness frameworks, alongside infection control strategies, is essential. Proactive investment in supportive environments, practical and coordinated guidance for families, continuity of movement-related services, and ethical population-level monitoring will be critical to strengthening system resilience and safeguarding children’s health during future public health emergencies.

## Supplemental Material

Supplemental material - Safeguarding Children’s Movement Behaviours During Public Health Crises: Lessons From a Global Rapid Scoping Review Supplemental material for Safeguarding Children’s Movement Behaviours During Public Health Crises: Lessons From a Global Rapid Scoping Review by Leigh M. Vanderloo, Taniya S. Nagpal, Richard Tyler, António L. Palmeira, Emily A. Bonisteel, Brianne A. Bruijns, Alissa J. Burnett, Ankhmaa Byambaa, Valerie Carson, Jordyn M. Cox, Audrey Elford, Rebecca B. Emori, Avril Johnstone, Rachel A. Jones, Sanne L. C. Veldman, Dona S. Yudasmara, Fariba Bannerman, & Anthony D. Okely in Environmental Health Insights.
