# Supplementary material for: Safeguarding Children’s Movement Behaviours During Public Health Crises: Lessons From a Global Rapid Scoping Review
Source: Environ Health Insights. 2026 Aug 14;20:11786302261479502. doi: 10.1177/11786302261479502 (PMC13477549; doi:10.1177/11786302261479502)
Supplement: Supplementary file 1 [file sj-pdf-1-ehi-10.1177_11786302261479502.pdf]

Supplementary Table 1. Study Characteristics.

Author	Country	Country Income	Sample Size	Age	Age Range	Male (%)	Female (%)	Movement Behaviours of Focus	Measurement Approach	Main Findings	Reported Changes in Movement Behaviours	BCW Component
Abe (92)	Japan	High	517		3 to 5	48.5	51.5	Physical Activity	Observation	For 25 m runs, participants aged 5 years were faster, while participants aged 3 and 4 years showed no significant differences. All participants aged 3–5 years showed no significant differences in the long jumps.		Intervention – Training
Abid (93)	Tunisia	Middle	100	8.66		52	48	Physical Activity, Sleep, Sedentary time	Parent/proxy report	Main findings showed that sleep quality was deteriorated during home confinement essentially for girls. Tunisian children decreased their PA levels and increased their ST during the confinement.	Physical activity decreased, Sleep decreased, Sedentary time increased	
Adibelli (94)	Turkey	High	597	9.87		44	56	Sleep, Sedentary time	Parent/proxy report	During the pandemic, 41.5% of the parents stated that their child gained weight, tendency to sleep of 34.2% and tendency to use the Internet of 69.3% increased.	Sleep increased; Sedentary time increased	
Aguilar-Farias (45)	Chile	High	3157	3.1		50.6	49.4	Physical Activity, Sleep, Sedentary time	Parent/proxy report	During early stages of the pandemic, time spent in PA decreased, recreational screen time and sleep duration increased, and sleep quality declined.	Physical activity decreased, Sleep had no change, Sedentary time increased	
Aguilar-Farias (95)	Chile	High	1727	2.9		52.1	47.8	Physical Activity, Sleep, Sedentary time	Parent/proxy report	Children reduced their PA, increased their screen time and their sleep quality declined during the early stages of the pandemic.	Physical activity decreased, Sleep decreased, Sedentary time increased	Policy – Communicaiton/Marketing
Aishworiya (96)	Singapore	High	593	8.68				Sleep	Parent/proxy report	Change in both maternal and paternal sleep duration positively correlated with change in child sleep duration among all children; preschoolers mirrored their mothers' sleep more closely. Parents who changed to working from home during the quarantine had the greatest gains in sleep during this period.		
Al-Mulla (97)	Qatar	High	144	8.1		57.7	42.3	Physical Activity, Sleep	Parent/proxy report	Most of the children had their bedtime and waketime shifted to later because of the quarantine. Total weekly average hours of PA significantly decreased with a greatest reduction for the school and after school durations.	Physical activity decreased; Sleep was unknown.	
Alanazi (46)	Saudi Arabia	High	1045	8.7				Physical Activity, Sleep, Sedentary time	Parent/proxy report	Only 1.8% of children met all components of the guidelines, compared to 3.4% in the before.		
Alanazi (98)	Saudi Arabia	High	1210	8.5				Physical Activity, Sleep, Sedentary time	Parent/proxy report	PA declined significantly and sleep increased. Girls had a greater increase in ST than boys.	Physical activity decreased, Sleep increased, Sedentary time was unknown.	Policy-Guidelines
AlHourani (99)	Jordan	High	477		6 to 12	51.4	49.6	Sedentary time	Parent/proxy report	Moreover, physical inactivity was increased significantly during the lockdown.	Sedentary time increased.	Intervention – Enablement
Ali (100)	Jordan	High	2781		1 to 6	53.1	46.9	Sedentary time	Parent/proxy report	During the pandemic the daily hours of Smartphone use among children were significantly higher than prior to the coronavirus-2019 (COVID-19) pandemic (2.12 vs. 1.7 h). More than half the children exceeded the recommended daily hours of use.	Sedentary time increased.	Intervention – Restriction
Alonso-	Spain	High	268	4.				Physical	Accelerome	Preschoolers showed a decrease in total PA (mean difference [MD] =43.3 min per day), sleep efficiency (MD	Physical activity decreased,	

Martínez (101)				28				Activity, Sleep, Sedentary time	ter; Parent/proxy report	=2.09%), an increase in ST (MD = 50.2 min per day).	Sleep decreased, Sedentary time increased.	
Amagliani (102)	Italy	High	507		5 to 13			Physical Activity	Parent/proxy report	Results show a remarkable decrease in children's PA during lockdown (88.9 vs 39.8% active children) associated with older age and low availability of outdoor spaces.	Physical activity decreased.	
Androutsos (103)	Greece	High	397	7.8		51.4	48.6	Physical Activity, Sleep, Sedentary time	Parent/proxy report	During the lockdown, children's/adolescents' sleep duration (>10h/night) and screen time (>3h/day) increased, while their PA decreased.	Physical activity decreased, Sleep increased, Sedentary time increased.	Intervention – Training
Arevalo (104)	Columbia	Middle	1139		4 to 12			Physical Activity	Parent/proxy report	It was found that during the mandatory isolation period: 75.2% of the children did not perform the minutes of PA recommended by the World Health Organization (WHO).		
Arundell (105)	Australia	High	436	8.7		58	42	Sedentary time	Self-report	There was little to no differences in reported in leisure time screen use.	Sedentary time was no change.	Intervention – Enablement, Environmental Restructuring, Training
Bacaro (106)	Italy	High	2361	8.1		51.4	49.6	Sleep	Parent/proxy report	In all group ages, late bedtime was observed (most of them after 9 p.m.). 59.4% of all children presented at least one clinical diagnostic criterion for childhood insomnia.		Intervention – Training
Bagherian (107)	Iran	Low	43660		8 to 17	35.6	64.4	Physical Activity	Self-report	32% of students reported doing less than one hour of exercise per day. Most students (72%) reported participating in PA at home.		
Bekelman (108)	USA	High	347	6.9		53	47	Physical Activity, Sleep, Sedentary time	Parent/proxy report	Compared with pre-pandemic, weekday screen time duration was higher during the pandemic. Sleep duration increased by 30 min among children who did not meet sleep recommendations pre-pandemic. Overall, no changes occurred in PA.	Physical activity had no change, Sleep increased, Sedentary time increased	Intervention – Training
Benzing (47)	Switzerland	High	57	10.44				Physical Activity	Accelerometer	Children who adhered to PA recommendations during stay-at-home restrictions had a better overall quality of life and psychological well-being than those who did not. It seems that the restrictions negatively impacted children's PA and quality of life.	Physical activity decreased	Intervention – Training, Environmental Restructuring, Enablement<br><br>Policy – Service Provision
Bergmann (109)	Multiple		2209		8 to 36 months			Sedentary time	Parent/proxy report	Caregivers reported that toddlers with no online schooling requirements were exposed to more screen time during lockdown than before lockdown.	Sedentary time increased	
Bingham (110)	United Kingd	High	949	10.5				Physical Activity	Self-report	There was a large reduction in children being sufficiently active during the first COVID-19 lockdown (28.9%) compared to pre-pandemic (69.4%).	Physical activity decreased	

	om											
Bisgin (111)	Turkey	High	30		8 to 11	100	0	Physical Activity, Sedentary time	Self-report	Results showed that there was a significant difference between the level of PA and sedentary of elementary students in the experimental and control groups. The intensity of the effect is equal to 0.58 and 0.66, respectively.		
Breidokienė (112)	Lithuania	High	306	9.65		47.1	52.9	Physical Activity, Sedentary time	Parent/proxy report	Results revealed that 57.5% of children exceeded the recommended maximum of 2 hours of recreational screen time per day, and 33.6% of the children did not meet the recommended guidelines of 60 min of PA per day.		Intervention – Education
Bruni (23)	Italy	High	4314		1 to 18	50.4	49.6	Sleep	Parent/proxy report	Found a significant delay in bedtime and risetime in all age groups.	Sleep increased.	
Brzęk (113)	Poland	High	1316		3 to 5	44.4	55.6	Physical Activity, Sleep, Sedentary time	Parent/proxy report	During the pandemic, the percentage of children meeting the recommendations for PA decreased even more. Children spent much more time in a sitting position before the restrictions. The pandemic caused an increase in sleep duration of 10–18%.	Physical activity decreased; Sleep increased; Sedentary time increased.	
Burkart (114)	USA	High	74		7 to 12			Physical Activity, Sleep, Sedentary time	Accelerometer; Parent/proxy report	During the pandemic ST increased and MVPA decreased. Sleep timing shifted later.	Physical activity decreased; Sleep had no change; Sedentary time increased.	
Burkart (115)	USA	High	231	9.8		53	47	Physical Activity, Sleep, Sedentary time	Parent/proxy report	Children's behaviours worsened at a greater rate following the pandemic onset compared to pre-pandemic trends. During pandemic spring, ST increased and MVPA decreased compared to change in previous springs.	Physical activity decreased; sleep increased; sedentary time increased.	Intervention – Environmental Restructuring
Cachón-Zagalaz (116)	Spain	High	837	6.22		50.2	49.8	Physical Activity, Sedentary time	Parent/proxy report	The time children devoted to sleep was directly proportional to the time they devoted to PA and indirectly proportional to the time they spent watching screens.		
Caldwell (48)	Canada	High	79	9.25		45	55	Physical Activity	Self-report	The average PAQ-C score was 2.70+/-0.48, PLAYself was 68.23+/-13.12, and PACES was 4.22+/-0.59 (mean +/- SD). Scores were below standard means (cognitive function, family relationships) or within normal limits.		
Caldwell (117)	Canada	High	844		7 to 12			Physical Activity, Sleep	Parent/proxy report	No differences from baseline to after the after-school program on PA and sleep disturbances.		Intervention – Education<br><br>Policy – Service Provision
Carroll (118)	Canada	High	310	5.7				Physical Activity, Sedentary time	Parent/proxy report	Screen time increased among 87% of children and PA decreased among 52% of children.	Physical activity decreased; Sedentary time increased.	
CartanyÃ-Hueso (119)	Spain	High	302	1.83		50	50	Sedentary time	Parent/proxy report	Two out of three children under 48 months living in Barcelona were daily exposed to smartphones and tablets during total lockdown due to COVID-19.		
Cassanello (120)	Spain	High	254		3 to 36			Sleep	Parent/proxy report	During confinement, infants and toddlers went to sleep later. More infants and toddlers showed longer sleep latencies during confinement.	Sleep decreased.	

					months							
Cellini (121)	Italy	High	299	7.96		54	46	Sleep	Parent/proxy report	During the lockdown, children showed a marked delay in sleep timing (later bedtime and rise time)	Sleep decreased.	
Censi (122)	Italy	High	1027		2 to 11			Physical Activity, Sedentary time	Parent/proxy report	78.1% of the children stopped their habitual PA. Children spent more time on devices, missed school and friends, being more bored and less creative.	Physical activity decreased; Sedentary time increased.	
Chambonniere (123)	France	High	6491		6 to 17	42	58	Physical Activity, Sedentary time	Parent/proxy report	Initially active children and adolescents decreased their PA more than those initially inactive, while those who met the sitting time recommendations increased more their sitting time during lockdown (p<0.001). The same applied to screen time.	Physical activity decreased; Sedentary time increased.	
Chau (124)	Hong Kong	High	1233	8.8		50	50	Sleep	Parent/proxy report	Primary school and secondary school participants assessed before COVID -19 a longer sleep latency.	Sleep increased	
Chen (125)	China	High	535	10.3		50	50	Sedentary time	Self-report	Schoolchildren spent significantly more time on the smartphone and social media but not gaming during the school suspension compared to the baseline.	Sedentary time increased	
Chioma (126)	Italy	High	490	6.6				Physical Activity, Sedentary time	Self-report	Overall time spent on electronic devices increased from 5-10 h in 2019 to 15-20 h per week. This likely was due to e-learning. Leisure time electronic use was the same at 5-10 h in 2019 and 2020. PA decreased in girls.	Physical activity decreased; Sedentary time increased.	Intervention – Education
Clarke (49)	United Kingdom	High	20		3 to 5			Physical Activity, Sleep, Sedentary time	Parent/proxy report	Most parents said that the pandemic made their child less active, more difficult to get them to sleep, and increased screen time.	Physical activity decreased, Sleep decreased, Sedentary time increased.	Intervention – Training<br>Policy – Guidelines
Contreras-Osorio (50)	Chile	High	90	11.45		51	49	Physical Activity	Self-report; Other	Current results consistently suggest the need for adequate levels of PA, physical fitness, and anthropometric parameters among the school-age population to promote healthy and adequate executive functions.		Intervention – Education, Environmental Restructuring<br>Policy – Environmental / Social Planning
Cristina Villodres (127)	Spain	High	899		10 to 14	46	54	Physical Activity	Self-report	A decrease in PA was observed during confinement in both boys and girls.	Physical activity decreased.	Intervention – Education, Training
Curatola (51)	Italy	High	205	9.9		44	56	Sleep	Self-report	Our survey showed that the COVID-19 pandemic led to major changes in the sleep-wake rhythm of children and adolescents: we observed a high prevalence (90%) of sleep-related difficulties in our population after 1 year of lockdown.		Policy – Fiscal, Environmental/Social Planning, Service Provision

de Sa (128)	Brazil	Middle	816		0 to 12			Physical Activity, Sleep, Sedentary time	Parent/proxy report	Most parents consider there was a reduction in the time that children spend practicing PA; increase in screen play time and family activities.	Physical activity decreased; Sleep increased; Sedentary time increased.	
Di Giorgio (52)	Italy	High	245	4.1		52	48	Sleep	Parent/proxy report	Children' sleep quality was, on average, less affected by the lockdown than mothers, although their sleep timing strongly shifted.	Sleep increased.	Policy – Service Provision
Diaz-Rodriguez (129)	Spain	High	478		0 to 6			Physical Activity, Sleep, Sedentary time	Parent/proxy report	The more children, the more screen viewing. Mothers indicated worse sleep during lockdown, but felt they promoted PA more. Parents working from home observed their child was on the screen more. Older children went to bed later.	Physical activity decreased; Sleep decreased; Sedentary time increased.	
Dunton (15)	USA	High	211	8.7		47	53	Physical Activity, Sedentary time	Parent/proxy report	Children were more likely to perform PA at home indoors or on neighborhood streets during the early- vs. pre-COVID-19 periods.	Physical activity levels decreased; Sedentary time increased.	
Eyler (130)	USA	High	245	8.1				Physical Activity	Parent/proxy report	Majority reported a decrease in PA levels.	Physical activity decreased.	Intervention – Education, Training, Enablement, Modelling
Farah (131)	Isarel	High	178	2.04		53	47	Sedentary time	Parent/proxy report	Parental pandemic stress was significantly associated with increased child screen use, parental unemployment was associated with screen use of the child.		Intervention – Education, Training
Fitzpatrick (132)	Canada	High	316	3.45		53	47	Sedentary time	Parent/proxy report	Results indicate that 64% of preschoolers used more than 2 h of digital media hours/day on average during the pandemic. A majority (56%) of children were also exposed to media within the hour before bedtime.		Intervention – Education
Francisco (133)	Multiple Countries		1480	9.1		53	47	Physical activity, Sleep, Sedentary time	Parent/proxy report	The results revealed an increase in children's psychological and behavioural symptoms, increased screen-time, reduced PA, and more sleep hours/night. Italian children presented less psychological and behavioural symptoms compared with Portuguese and Spanish children.	Physical activity decreased, Sleep increased, Sedentary time increased.	
Ganzar (134)	USA	High	168		8 to 11			Physical Activity, Sedentary time	Accelerometer	There was a significant decrease in mean daily MVPA and an increase in ST.	Physical activity decreased; Sedentary time increased	Intervention – Education
GarcÃa-Alonso (135)	Spain	High	110	5.77		49	51	Physical Activity, Sleep, Sedentary time	Parent/proxy report	Children performed more MVPA on weekdays, had higher ST and slept fewer hours during the pandemic than before.	Physical activity increased, Sleep decreased, Sedentary time increased	Intervention – Education
Ghanamah (53)	Isarel	High	382		5 to 11	51	49	Physical Activity, Sleep, Sedentary time	Parent/proxy report	The findings of the current study indicate, significantly, changes in the children's daily routines during the pandemic outbreak and the lockdowns including increase of daily spending hours in engagement with screen, decrease in PA and greater sleeping hours per day.	Physical activity decreased; Sleep increased; Sedentary time increased.	Intervention – Training, Restrictions, Environmental

												Restructuring
Gilbert (136)	USA	High	144	8		56	44	Physical Activity, Sedentary time	Parent/proxy report	Most parents reported a decrease in child PA and an increase in child ST.	Physical activity decreased; Sedentary time increased.	Intervention – Education
Hanbazaza (137)	Saudi Arabia	High	280	10		51	49	Physical Activity, Sedentary time	Parent/proxy report	PA did not significantly change before and during COVID-19 curfew. However, the curfew led to an increase in the number of hours spent watching TV and playing video games per day.	Physical activity had no change; Sedentary time increased.	Intervention – Education, Environmental Restructuring<br><br>Policy – Environmental/Social Planning
Her (54)	South Korea	High	186	10.1		56	44	Sleep, Sedentary time	Parent/proxy report	Compared to 2020, smartphone screen time, tablet PC screen time per day and smartphone usage frequency per week increased significantly in 2021. There was no significant change in children's sleep problems.	Sleep had no change; Sedentary time increased.	Intervention – Education<br><br>Policy – Environmental/Social Planning
Hossain (138)	Bangladesh	Middle	63	4.5		55	45	Physical Activity, Sleep, Sedentary time	Parent/proxy report	The study inferred that the prevalence of overweight//obesity and underweight has disproportionately risen, and the degree of BMI has been substantially influenced by several demographic and socioeconomic factors, including eating habits and PA.	Physical activity decreased, increased sedentary time	
Houser (87)	Canada	High	160	11.1		52	48	Physical Activity	Self-report	28% of children did not meet the PA guidelines pre-pandemic, while 66% fell below threshold during the pandemic.	Physical activity decreased	Policy – Fiscal
Hurter (55)	United Kingdom	High	517		8 to 18	48	52	Physical Activity, Sedentary time	Accelerometer	During lockdown, MVPA was 38.4 ± 24.3 min/day; ST was 849.4 ± 196.6 min/day; mean ± SD. PA increased significantly upon return to school.	Physical activity decreased; Sedentary time increased	Policy – Environmental/Social Planning
Huster Siegle (56)	Brazil	Middle	916		3 to 12	52	48	Physical Activity, Sedentary time	Parent/proxy report	Social distancing promoted a decrease in activities involving PA and an increase in screen time among Brazilian children.	Physical activity decreased; Sedentary time increased	Policy – Environmental/Social Restructuring
Hyunshik (139)	Japan	High	504	3.6		53	47	Physical Activity, Sedentary time	Accelerometer; Parent/proxy report	Compared to the pre-COVID-19 period, preschool-aged children engaged in less weekday MVPA but engaged in more screen time during the COVID-19 pandemic.	Physical activity decreased; Sedentary time increased	Intervention – Training
Idoiaga (140)	Spain	High	250	7.1				Physical Activity, Sedentary time	Self-report	It appears that children are in urgent need of PA contact with the outdoors, along with the opportunity to maintain contact with their friends, classmates, loved ones, and teachers. It is also clear that due to the lockdown situation, it is not possible to meet these needs in a traditional way.		

Ijaz (141)	Pakistan	Middle	102		2 to 10	39	61	Sleep, Sedentary time	Parent/proxy report	There was marked increase in their screen time/per day from <2hrs before pandemic in 63 (61.8%) to >2hours during pandemic in 81 (79.4%). As a result, sleep habits were significantly affected during the pandemic, especially in the domains of bedtime, sleep behaviour and morning wake up.	Sleep had no change, Sedentary time increased	Intervention – Restriction
JÃ¡uregui (142)	Mexico	Middle	631	3.3		53	47	Physical Activity, Sleep, Sedentary time	Parent/proxy report	During the COVID-19 lockdown, toddlers and preschoolers in Mexico performed less PA, more ST and had lower sleep quality.	Physical activity decreased, Sleep decreased, Sedentary time increased	Intervention – Restriction<br>Policy – Environmental/Social Planning
James (143)	United Kingdom	High	1333	9.9				Physical Activity, Sleep, Sedentary time	Self-report	PA improved during school closures achieving 60 minutes of activity per day and 10% were more likely to meet sleep recommendations. Screen time also increased.	Physical activity increased, Sleep increased, Sedentary time increased	Intervention – Incentivization
Jovanovic (144)	Croatia	High	1370	12.7		47	53	Physical Activity, Sleep, Sedentary time	Self-report	COVID-19 lockdown has caused an increase in the proportion of overweight/obesity among Croatian school children who changed their lifestyle habits towards being les PA, spending more time using screen-based media.		Intervention – Education
Kaditis (37)	USA	High	845	9.9		55	45	Sleep	Parent/proxy report	Identified a shift of bedtime and wake-up times to later hours with unchanged sleep duration for half of them and either increased or decreased sleep length for the rest of them.		Intervention – Restrictions
Kahn (145)	USA	High	572	5.9		54	46	Sleep	Other	Infants of mothers in home confinement woke up 9 minutes later, and slept 14 minutes longer per night, compared to infants of essential workings working as usual mothers. Infants of mothers in home confinement had later sleep offset times and longer night-time sleep durations, compared to infants of mothers working as usual.		Intervention – Restrictions, Modelling
Kamaleddine (57)	Lebanon	Middle	383	5.3				Sedentary time	Parent/proxy report	Children using smartphones and TV/PlayStation/Xbox for more than 2 hours were more prone to sleep problems. 2. From this study, children's eating habits while using a screen were related to excessive screen time mainly with smart phones, sleep problems, and eating unhealthy food.		Intervention – Modelling<br><br>Policy – Guidelines
Khozaei (146)	Iran	Middle	276		10 to 12			Physical Activity	Parent/proxy report	Study sheds light on parental role in children's wellbeing and mental health during COVID-19 pandemic.		
Kim (147)	Japan	High	217	9.16				Physical Activity, Sleep, Sedentary time	Parent/proxy report	Weight gain increased, less time in PA and increase in media usage. Children's sleep problems time on tablet and behavioural problems were related to parental stress. Sleep was also associated with smartphone and tablet use.		
Knowland (148)	United Kingdom	High	288	8		52	48	Sleep	Self-report; Parent/proxy report	Current data show older children and adolescents were more likely to show a delay in sleep patterns over the course of lockdown.		Intervention – Education, Training
Konstantinou (149)	Greece	High	1509	9.6		52	48	Physical Activity, Sedentary time	Parent/proxy report	Children's PA decreased compared to pre-lockdown, while screen time increased in the post-lockdown period, with 25% of children spending 4-7 hours/day in front of screens vs. 10% in the pre-lockdown period.	Physical activity decreased; Sedentary time increased.	
Koran (150)	Cyprus	High	361		36 to 72 mont	38	62	Sedentary time	Parent/proxy report	Found a decrease in the use of mobile technology post-pandemic.		Intervention – Education,

					hs							Training, Restriction
Kovacs (58)	France	High	24302	12		52	48	Physical Activity, Sedentary time	Self-report; Parent/proxy report	European school-aged children had a low amount of PA and high levels of ST during winter lockdown. Less than 10% met the WHO PA recommendation which is a significant public health concern.		Intervention – Training<br><br>Policy – Environmental/Social Planning
Kracht (151)	USA	High	1836	3.9				Physical Activity, Sleep, Sedentary time	Self-report; Parent/proxy report	Household chaos was related to preschooler movement behaviours. Many mothers reported their household was moderately chaotic to very chaotic. Preschoolers within these households were less PA, slept less and engaged in more screen-time.		
Krijger (152)	Netherlands	High	1214	10.9		49	51	Physical Activity	Self-report; Parent/proxy report	Less physically active children reported having less social interaction with friends since the COVID-19 pandemic.		Intervention – Training
Kuhn (153)	USA	High	75		5 to 14	37	63	Physical Activity, Sleep, Sedentary time	Accelerometer	Pre-pandemic to early-pandemic changes in children's PA behaviour differed by age group. Compared to younger children, middle schoolers declined in weekday MVPA and increased in both ST and sleep times.	Physical activity decreased, Sleep increased, Sedentary time increased	
Kurz (154)	Germany	High	362	7		48	52	Physical Activity, Sleep, Sedentary time	Self-report	Participating in MVPA compared with before the pandemic. This higher proportion may be attributed to children spending more time being sedentary and less time visiting parks during the pandemic.	Physical activity had no change, Sleep had no change, Sedentary time unknown	
Kw (155)	South Korea	High	246		6 to 12	53	47	Physical Activity	Self-report; Parent/proxy report	Children at joint-use parks during the pandemic was due to the COVID-19 order, changes in PA behaviour of children, and impacts of the pandemic on guardians of children.		
Lafave (59)	Canada	High	238	4.1		49	51	Physical Activity, Sedentary time	Accelerometer; Parent/proxy report	We posit the decline in the number of children and number of PA.	Physical activity decreased; Sedentary time increased	Intervention – Training, Education<br><br>Policy – Communications/Marketing, Guidelines
Lanza (60)	USA	High	1210		1 to 12	55	45	Physical Activity	Parent/proxy report	Increase in vigorous intensity PA during the pandemic than before, as well as a decline in the number of PA children in parks during the pandemic versus before.	Physical activity decreased	Policy – Communications/Marketing, Environmental/Social Planning
Lee (61)	South Korea	High	217	9		56	44	Sedentary time	Parent/proxy report	Lower household income was associated with greater screen time (ST).	Sedentary time increased	Policy – Service Provision,

												Guidelines
Li (156)	Canada	High	256	5.5		53	47	Sedentary time	Parent/proxy report	COVID-19 were associated with a negative impact on the health behaviours of Canadian children, associated with a decrease in outdoor time and an increase in screen time during the COVID-19 pandemic.	Sedentary time increased	Policy – Guidelines
Lima (157)	Brazil	Middle	105		8 to 10	46	54	Sleep	Parent/proxy report	Percentage of children with sleep disorders and sleep bruxism increased during the COVID-19 pandemic.	Sleep decreased	Intervention - Education
Liu (158)	China	High	1619	5.4		49	51	Sleep	Parent/proxy report	Later bedtimes and wake times, longer nocturnal and shorter nap sleep durations, comparable sleep duration in 24-hour period, fewer sleep disturbances.	Sleep unknown	
Loo (159)	Singapore	High	296		7 to 17	41	59	Physical Activity, Sedentary time	Self-report	Overall, only 4% of primary school students met the daily PA recommendations. 48% of primary school students did aerobic activities. A major concern was high ST as 73% of participants exceeded 2 hours of daily ST.		
Lopez-Avmes (160)	Spain	High	234	8 to 17		54	46	Physical Activity	Parent/proxy report	146 parents believed their child's PA was insufficient.		
Lopez-Bueno (161)	Spain	High	860	9.6		51	49	Physical Activity, Sleep, Sedentary time	Parent/proxy report	Decreased PA, Increased screen time (ST) and sleep time.	Physical activity decreased; Sleep increased; Sedentary time increased	
Lopez-Gil (162)	Brazil	Middle	495	10.7		56	44	Sleep	Parent/proxy report	12.3% were not meeting sleep guidelines and 48.8% of participants reported sleep-related problems.		
Lopez-Gil (163)	Brazil	Middle	1099	10.7		46	54	Physical Activity, Sleep, Sedentary time	Parent/proxy report	PA and ST decreased during lockdown, while sleep duration increased. Portion of sample meeting the overall 24-h guidelines was very low before lockdown and even worse during the lockdown.	Physical activity decreased, Sleep increased, Sedentary time decreased	
Luszczki (164)	Poland	High	1016	10.5		49	51	Physical Activity, Sleep, Sedentary time	Self-report; Parent/proxy report	Decreased PA during the pandemic, decrease in sleep times on school days, increased video game time, but decreased phone usage during the pandemic on a regular day.	Physical activity decreased, Sleep had no change, Sedentary time unknown	
MacKenzie (165)	Canada	High	85	8.1		40	60	Sleep	Parent/proxy report	While some parents perceived their child’s sleep quality improved during the COVID-19 pandemic (14.1%), many parents perceived their child’s sleep had worsened (40.0%).	Sleep decreased	
Markovic (166)	Switzerland	High	864	1.5		48	52	Sleep	Parent/proxy report	An acute decrease in sleep quality was found in both groups of children. However, at follow- up assessments, this effect rebounded to the level reported for the period before the confinement.	Sleep decreased	Intervention – Restriction, Education
Maximova (62)	Canada	High	1095		9 to 12	49	51	Physical Activity, Sleep, Sedentary time	Self-report	A majority of students reported declines in PA, having late bed/wake-up times, and modest improvements in mental health and wellbeing. Many students reported increases rather than decreases in screen time and snacking.	Physical activity decreased, Sleep increased, Sedentary time increased	Policy – Service Provision
McNicholas (167)	Australia	High	1371	6.8				Physical Activity, Sleep, Sedentary time	Parent/proxy report	Increased ST by children’s and families, and slight increase in PA, amount of food consumed, and meals and snacks eaten during the height of COVID-19. Little change in reported sleep.	Physical activity increased, Sleep had no change, Sedentary time increased	

Medrano (63)	Spain	High	291	12.1		52	48	Physical Activity, Sleep, Sedentary time	Accelerometer; Self-report	PA and screen time worsened during COVID-19, with prevalence of screen usage increasing. Sleep time increased both on weekends and during the weekend days.	Physical activity decreased, Sleep increased, Sedentary time increased	Policy – Environmental/Social Restructuring
Mitra (64)	Canada	High	693		5 to 11			Physical Activity	Parent/proxy report	Living in houses was correlated with more outdoor activities, and closeness to major roads a barrier. Low compliance to movement guidelines.		Intervention – Environmental Restructuring<br><br>Policy – Environmental/Social Planning
Mitra (65)	Canada	High	315		5 to 17			Physical Activity, Sleep, Sedentary time	Parent/proxy report	Not maintaining healthy movement behaviours was associated with low subjective well-being, they were also more likely to spend more time on screens. No impact of sleep duration.	Physical activity unknown, Sleep had no change, Sedentary time increased	Intervention – Environmental Restructuring<br><br>Policy – Environmental/Social Planning
Molu (168)	Turkey	High	122	4.8		48	52	Sleep, Sedentary time	Parent/proxy report	More time watching TV and using smart phone devices since the pandemic. More ST was associated with temperament and sleep problems.	Sleep unknown, Sedentary time increased	
Monteiro (169)	Portugal	High	193	3.5		56	44	Sedentary time	Parent/proxy report	The majority of all the participant parents (71%, n = 137) agreed that exposure time to TV had increased during the confinement.	Sedentary time increased	
Moore (26)	Canada	High	1472	11.5		46	54	Physical Activity, Sleep, Sedentary time	Parent/proxy report	While most children and youth were meeting sleep recommendations for their age (59.3%), most were not meeting the PA or screen time guidelines (only 14.3% and 25.6% meeting the guidelines, respectively).		
Moore (170)	Canada	High	1472	8.1		46	54	Physical Activity	Parent/proxy report	Children and youth experienced a significant decline in all PA.	Physical activity decreased	
Nathan (88)	Australia	High	157	6.9				Physical Activity	Parent/proxy report	Overall, weekly minutes of total PA did not change from before to during COVID-19. However, the frequency and duration (total and home-based) of unstructured PA significantly increase.	Physical activity had no change	Policy – Fiscal
Neshteruk (171)	USA	High	51	9.7				Physical Activity, Sedentary time	Parent/proxy report	No consensus on PA levels. Reported increase in screen time levels.	Physical activity unknown; Sedentary time increased	Intervention – Training
Ng (172)	China	High	89	4.4		72	28	Physical Activity, Sleep, Sedentary time	Accelerometer; Parent/proxy report	Results suggested that children spent more time sleeping (8% increase) and in MVPA (16% increase), with less time spent in ST (9% decrease).	Physical activity increased; Sleep increased; Sedentary time decreased	
Nystrom	Sweden	High	81	4		58	42	Physical	Parent/prox	PA increased during the pandemic, and screen time increased during the pandemic.	Physical activity increased;	

(173)	en							Activity, Sedentary time	y report		Sedentary time increased	
Oflu (66)	Turkey	High	253	6.3		53	47	Sedentary time	Parent/proxy report	It was found that the ratio of children with screen time of ≥1 hour during the pandemic was significantly higher than before the pandemic.	Sedentary time increased	Intervention – Education, Training, Restriction
Okely (174)	Australia	High	948	4.4		51	49	Physical Activity, Sleep, Sedentary time	Accelerometer; Parent/proxy report	Children decreased their nap amounts by an average of 19 minutes a day, spent 81 minutes less outdoors a week. There were significant increases in time spent sitting and consuming television.	Physical activity decreased; Sleep decreased; Sedentary time increased	
Olive (175)	Australia	High	2365	9		19	81	Physical Activity, Sleep	Parent/proxy report	1 in 6 parents reported that their children started having sleeping problems and had behavioural issues because of it. It was also found that kids were participating in less MVPA for smaller amounts of time on fewer days.	Physical activity decreased; Sleep decreased	
Ophir (176)	Isarel	High	291		5 to 12			Sedentary time	Parent/proxy report	Increase in use of screens for entertainment among all age groups, increase in educational use of screens in all age groups.	Sedentary time increased	
Ostermeier (177)	Canada	High	27		10 to 13	15	85	Physical Activity	Parent/proxy report	Study found there were changes to participant PA as there was increases in the amount of time spent outside and more altered environments.		
Ostermeier (67)	Canada	High	95	11		51	49	Physical Activity, Sedentary time	Parent/proxy report	Significant changes in PA and screen time behaviours were identified between gender, SES, and facility use groups. Overall, COVID-19 protocols have negatively influenced children's PA and screen time.	Physical activity decreased; sedentary time increased	Policy – Environmental/Social Planning
OzturkEyimaya (178)	Turkey	High	1159		6 to 13	47	53	Sedentary time	Parent/proxy report	Families have tried to establish ground rules related to screens although majority still saw an increase in use amounting to 6.42 hours per day.	Sedentary time increased	Intervention – Education, Training, Restrictions
Pasi (179)	India	Middle	278		1 to 12			Sedentary time	Self-report	Television was the most common used media followed by cell phones. Children aged 5-12 watched more television than 1-4. Majority of children used screens for educational purposes.		Intervention – Training, Restrictions
Pedrotti (180)	Brazil	Middle	513	1.5		50	50	Sedentary time	Parent/proxy report	Found increased media duration among mothers and toddlers, and mothers' media use duration and mothers' intention to offer media significantly explained children's media use duration.	Sedentary time increased	
Peliccciari (181)	Brazil	Middle	68	10.1		44	56	Physical Activity, Sedentary time	Self-report	Was mainly looking at weight gain during COVID -19 but one of the biproducts of those who were gaining weight was that they did not do much PA and had high levels of daily ST.		
Pelletier (68)	Canada	High	23		7 to 12	61	39	Physical Activity	Parent/proxy report	Children had increases in unstructured activity and a decrease in structured PA which many felt was a positive change.	Physical activity increased	Intervention – Environmental Restructuring
Perez (69)	USA	High	321	9.8		53	47	Physical Activity	Parent/proxy report	Shelter in place mandates restricted children's PA and those who had access to outdoor spaces and equipment had higher levels of PA compared to those without.		Intervention – Environmental<br><br>Policy –

												Fiscal, Environmental/Social Planning
Picca (70)	Italy	High	1688		1 to 10	51	49	Sleep	Parent/proxy report	During confinement parents found that there were higher frequencies of their children sleeping as compared to before COVID-19.	Sleep increased	Intervention – Training, Enablement<br><br>Policy – Regulation, Service Provision
Planinsec (89)	Slovenia	High	3936	8.8		53	47	Physical Activity	Parent/proxy report	Decline in children's PA during COVID-19 and big decline in extracurricular sports activities which before COVID-19 had been participated in by 72.2% of kids while during COVID-19 as many as 83.5% of children did not participate in extracurriculars.	Sedentary time increased	Policy – Regulation
Pombo (182)	Portugal	High	2159		0 to 12	52	48	Physical Activity, Sedentary time	Parent/proxy report	During confinement there was a decrease in children's PA and screen time. Boys engaged in more screen time than girls and boys "played" more with PA than girls.	Physical activity decreased, Sedentary time increased	
Pombo (183)	Portugal	High	2159		0 to 12	52	48	Physical Activity	Parent/proxy report	Found that boys and girls didn't differ in their PA percentage but that children being younger, having a big outdoor space, having other children in the household, and having at least one adult free from working from home were significantly more active.		
Poulain (71)	Germany	High	152		1 to 10	100	0	Physical Activity, Sedentary time	Self-report	Throughout COVID-19 lockdowns, children's PA along with their physical and mental wellbeing. Coping with homeschooling decreased as well and there were increases in screen time throughout both lockdowns.	Physical activity decreased; Sedentary time increased	Intervention – Enablement
Poulain (184)	Germany	High	285	5.6		53	47	Physical Activity, Sedentary time	Parent/proxy report	The frequency of playing outside increased but frequency of indoor sports and activity, motivation to do schoolwork, playing boardgames and PA decreased. Higher media use was also found specifically in lower socioeconomic households.	Physical activity decreased; Sedentary time increased	Policy – Environmental/Social Planning
Reece (185)	Australia	High	16177		4 to 18			Physical Activity, Sedentary time	Parent/proxy report	Since the COVID- 19 restrictions, most respondents reported that children's PA levels had decreased (a lot: 31%, a little: 39%). Most children's screen time increased (a lot 48%, a little 37%).	Physical activity decreased; Sedentary time increased	
Reséndiz-Aparicio (72)	Mexico	Middle	4000		4 to 15			Physical Activity, Sleep, Sedentary time	Parent/proxy report	The use of screens increased 30-80% in over 65% of children and 20.3% reported sleeping disorders; 23.6% could perform PA.	Physical activity unknown, Sleep unknown, Sedentary time increased	Policy – Environmental/Social Planning
Ribner (186)	Multiple		2516	5.7		51	49	Sedentary time	Parent/proxy report	On average, children engaged with screens more than 50 minutes more during the pandemic than before. Parent report of child total screen time increased across all countries except China.	Sedentary time increased	Intervention – Enablement
Richards (187)	United Kingdom	High	23	12		61	39	Physical Activity, Sedentary time	Self-report; Parent/proxy report	Stay-at-home mandates resulted in perceived reductions in PA and increases in ST within the home.	Physical activity decreased; Sedentary time increased	Intervention – Training, Restrictions
Rocka (188)	Poland	High	3127	12.1		53	47	Physical Activity, Sedentary time	Parent/proxy report	During a routine weekday, most children (71%) spent >4 h on educational activities using electronic devices. Parents reported that the time their children spent performing daily PA during a regular weekday was 2.08 ± 1.8 h, with a median of 1.5 h.		Intervention – Restrictions

Salway (189)	United Kingdom	High	1689	10.9		48	52	Physical Activity, Sedentary time	Accelerometer	Children’s MVPA was lower post-COVID-19 by around 7–8 min per day when compared to data collected in the same schools, in the same way, prior to the COVID-19 pandemic. Average daily ST was higher for children by nearly half an hour.	Physical activity decreased; Sedentary time increased	
Sanmiguel-Rodriguez (90)	Multiple		2316	7.7		52	48	Physical Activity, Sleep, Sedentary time	Self-report; Parent/proxy report	The findings of this study indicate that children residing in Europe showed higher values in the time (expressed in minutes) spent doing PA (M = 36.97 ± 34.36) and hours of sleep (M = 9.60 ±7.21); while in Libero-America, there were longer times in the use of video consoles, computers, mobile phone.		Intervention – Training<br><br>Policy – Regulation
Saxena (73)	India	Middle	1237	11.88		59	41	Physical Activity, Sedentary time	Self-report	Decrease in time spent on outdoor activity (from 8.5 hours/week pre-COVID-19 to 1.6 hours/week during lockdown). Increase was noted in screen time spent on digital devices (from 6.2 hours/week to 19.8 hours/week) and watching television (from 12.2 hours/week to 13.4).	Physical activity decreased; Sedentary time increased	Policy – Guidelines
Schmidt (190)	Germany	High	1711	10.5		50	50	Physical Activity, Sedentary time	Parent/proxy report	We found that sports activity declined whereas recreational screen time increased. However, a substantial increase in habitual PA leads to an overall increase in PA among children and adolescents in Germany.	Physical activity increased; Sedentary time increased	
Schnaiderman (74)	Argentina	Middle	267	11.1		47	53	Physical Activity, Sleep, Sedentary time	Parent/proxy report	The median sleep time on weekdays increased by 30 minutes, and the median screen time for entertainment on weekdays increased by 3 hours. Time dedicated to PA did not change, but the type of activities did.	Physical activity had no change, Sleep increased, Sedentary time increased	Policy – Environmental/Social Planning
Segre (75)	Italy	High	82	10.4		54	46	Sleep	Self-report; Parent/proxy report	50 respondents (61 %) reported changes in sleep pattern: most of them had difficulties in falling asleep and woke up many times during the night.	Sleep unknown	Policy – Environmental / Social Planning
Seguin (191)	Canada	High	75		6 to 12			Sedentary time	Parent/proxy report	Children's screen time increased during the pandemic compared to the pre-pandemic period. On average children's time spent viewing a screen increased from over 2 h per day (average=2.6 h) to nearly 6 h per day (average=5.9 h) during the pandemic.	Sedentary time increased	
Shinomiya (76)	Japan	High	2312		1.5 to 2.5	50	50	Sleep	Parent/proxy report	There was no significant difference between 2019 and 2020 regarding the wake-up times, bedtimes, or nocturnal sleep patterns of the children.	Sleep had no change.	Policy – Environmental/Social Planning.
Shneor (192)	Isarel	High	19		8.5 to 12	100	0	Physical Activity, Sleep	Accelerometer	During restrictions, children spent significantly less time outdoors, were less active, and spent less time engaged in MVPA. Sleep duration was not significantly different, but bedtime and wake time shifted to a later time during restrictions.	Physical activity decreased; Sleep increased.	
Shrestha (193)	Nepal	Middle	630	7		50	50	Sedentary time	Parent/proxy report	The median screen time before the COVID- 19 pandemic was 1.0 hours (mean rank=275.8) which increased to 2.2 hours (mean rank=116.6) during the pandemic (p<0.001).	Sedentary time increased.	
Siachpazidou (194)	Greece	High	482	8.1		53	47	Physical Activity, Sleep, Sedentary time	Parent/proxy report	Almost half of all families reported that the quarantine period negatively impacted PA and quality of sleep. Web use showed an increase, with the children over-spending extracurricular time in web activities	Physical activity decreased; Sleep unknown; Sedentary time increased.	
Siegle (195)	Brazil	Middle	816		0 to 12	50	50	Physical Activity	Parent/proxy report	The results show that having large external space at home and age influence the percentage of PA performed by children during social distancing due to COVID-19.		
So (196)	China	High	1016	12.3		32	68	Physical Activity, Sleep, Sedentary time	Accelerometer; Parent/proxy report	We objectively demonstrated that children had lower PA levels, longer sleep duration, and longer screen time during school closures (March 2020–April 2020) compared with the pre-pandemic time (September 2019–January 2020).	Physical activity decreased; Sleep increased; Sedentary time increased.	

Stienwandt (197)	Canada	High	708		2 to 8			Sedentary time	Parent/proxy report	Total time spent supervising child screen activities was estimated at M =16.48 (SD =11.06) hours per week; combined screen time based on a specific activity was M =17.34 (SD =14.22) hours per week.		
Štveráková (198)	Czech Republic	High	98	10		43	57	Physical Activity	Accelerometer; Self-report	Findings demonstrate a significant decline in PA in children during COVID-19 compared to pre-COVID-19. The average number of steps was 7.767 steps/day (boys = 9.255; girls = 6.982).	Physical activity decreased	
Sum (199)	Singapore	High	604	7.1		49	51	Physical Activity, Sleep, Sedentary time	Self-report; Parent/proxy report	33% of the primary school aged children and 25% of the preschool aged children did not play outside. Children reported less than 10 hours of sleep per night during lockdown, even on weekends or public holidays.	Physical activity decreased, Sleep unknown, Sedentary time increased	
Sundqvist (200)	Sweden	High	240		4 to 6	48	52	Sedentary time	Parent/proxy report	Children watched 643 mins of television per week, two thirds communicated through video chat, for a mean of 65 minutes a week, 72% played child friendly video games for 205 mins per week. No age or gender differences.	Sedentary time increased	Intervention – Modelling
Susilowati (77)	Indonesia	Middle	951		2 to 6			Sedentary time	Parent/proxy report	Preschool aged children spent at least an hour on the screen, which was mostly the phone, followed by tv, and then computer.		Intervention – Environmental Restructuring
Szpunar (78)	Canada	High	9	7.3				Physical Activity	Parent/proxy report	To deal with inactivity children said the best facilitator was sending kids outside. Parents were willing to let their kids get back to sports and outdoor play once deemed safe. Students missed their friends and other individuals in sport such as coaches.		Intervention – Environmental Restructuring
Tandon (79)	USA	High	1000	10.8		53	47	Physical Activity	Self-report; Parent/proxy report	20% of children indicated 60 minutes of daily PA. On average children were active for 3 days per week for 60 minutes and had 4 hours of screen time.		Policy – Environmental/Social Planning
Thiab (201)	Jordan	High	230	9		55	45	Physical Activity, Sleep, Sedentary time	Self-report; Parent/proxy report	PA decreased, and screen time and sleep hours increased.	Physical activity decreased; Sleep increased; Sedentary time increased.	
Tulchin-Francis (80)	USA	High	1296		5 to 12	50	50	Physical Activity	Parent/proxy report	Significant decline in MVPA while light activity stayed the same, however this was not as impactful in preschoolers than older children.	Physical activity had no change.	Policy – Guidelines
Ueno (202)	Japan	High	959		6 to 11	51	49	Sleep, Sedentary time	Parent/proxy report	Sleep behaviours were not different between children with or without behavioural problems, body composition was associated with screen time during the closures.		
Ustuner-Top (81)	Turkey	High	1040	9.6		51	49	Sleep	Parent/proxy report	55% experienced sleep disturbances, including bedtime resistance, delay to sleep, and sleep duration.		Intervention – Education, Training<br><br>Policy – Service Provision
Valadez (203)	Italy	High	325		5 to 14			Physical Activity	Parent/proxy report	No differences in PA and well-being amongst communities. Parents said there was more time spent on homework than play.		
Vandoni	Italy	High	37	1		75	25	Physical	Self-report	An online training on PA improved physical fitness, BMI, and waist circumference reduction.		Interventio

(204)				1				Activity				n - Training
Ventura (205)	Spain	High	3464		6 to 11			Physical Activity, Sleep	Parent/proxy report	Increase in delay to bedtime and inadequate sleep hours, odds of inadequate PA level increased in the pandemic.	Physical activity decreased; Sleep unknown.	
Verga-Perona (204)	Spain	High	37	2.5		55	45	Physical Activity, Sedentary time	Accelerometer	Toddlers reported high levels of total PA and MVPA during childcare hours. Girls and boys had similar activity patterns. Classroom density was associated with ST.		
Vukovic (206)	Serbia	High	450		5 to 13	51	49	Physical Activity, Sedentary time	Parent/proxy report	Children who were active before the pandemic remained active. ST was associated with poor mental health outcomes.	Physical activity had no change	
Walker (207)	United Kingdom	High	47	11.5		55	45	Physical Activity	Self-report	Early pandemic the lockdown was a short-lived adventure, access to facilities to be active were reduced, and parenting responsibility increased.		
Wang (208)	Taiwan	High	21761	5.5		54	46	Sedentary time	Parent/proxy report	More than 1-2 hours per day were spent on the screen.		
Wang (209)	China	High	6017		6 to 10			Physical Activity, Sedentary time	Parent/proxy report	Screen time was positively related to mental health problems, and physical exercise was negatively related.		
Wearick-Silva (210)	Brazil	Middle	577		0 to 12			Sleep	Parent/proxy report	Increase in sleep concerns around initiating sleep or maintaining sleep in 58% of children ages 0-3 and 34% of children 4-12 years.		
Wessely (211)	Germany	High	1238	8.1		53	47	Physical Activity	Observation	There was a difference in achievement in physical performance skills (e.g., long jump) among children from low-income backgrounds or who experienced social burdens including having a higher body mass index.		Intervention – Enablement
Wilson (212)	USA	High	261		9 to 13			Physical Activity	Self-report	Students reported less time being active during recess and overall.		
Widyastri (82)	Thailand	High	3735		6 to 17			Physical Activity	Self-report; Parent/proxy report	PA decreased; girls had lower activity levels than boys.	Physical activity decreased.	Policy – Guidelines
Wunsch (213)	Germany	High	1711	10.3		51	49	Physical Activity, Sedentary time	Self-report	Pre-pandemic behaviours were predictive of behaviours during the pandemic. There was an increase in ST.	Physical activity had no change; Sedentary time increased.	
Xiang (214)	China	High	2423	11.7		48	52	Physical Activity, Sedentary time	Parent/proxy report	Longer screen time was associated with poor psychological symptoms and engaging in more PA led to improved psychological symptoms.		
Yelizarova (83)	Ukraine	Middle	1091		6 to 18	54	46	Physical Activity	Parent/proxy report	PA decreased by 12% in 201 compared to before the pandemic. The proportion of children meeting MVPA guidelines decreased.	Physical activity decreased.	Policy – Guidelines, Regulation
Zachurzok (91)	Poland	High	177	12.8		46	54	Physical Activity, Sleep, Sedentary time	Self-report; Parent/proxy report	The number of children meeting MVPA guidelines declined since pre-pandemic, more children slept more than 8 hours daily, and screen time increased.	Physical activity decreased; Sleep increased; Sedentary time increased.	Policy – Regulation
Zafar (84)	Pakistan	Middle	923		0 to 12			Sedentary time	Parent/proxy report	Prior to the pandemic most children were grouped as having screen time for 2 hours daily whereas during lockdown most viewed screens for over 2 hours.	Sedentary time increased	Policy – Guidelines,

												Fiscal, Service Provision
Zengin (85)	Turkey	High	309	10.3		52	48	Sleep, Sedentary time	Parent/proxy report	About 4 hours per day was spent using a tablet or smartphone, majority of the children slept for 8-10 hours suggested their sleep patterns have become more irregular including a later bedtime.		Intervention – Education, Training<br><br>Policy – Service Provision
Zhang (215)	China	High	9979	11.6		51	49	Physical Activity	Parent/proxy report	The results suggested that the MVPA of students has dropped badly, compared with the results of previous studies investigated in normal times. In addition, the PA level had a significantly positive impact on the mood states of children and adolescents during the COVID-19 epidemic.	Physical activity levels decreased	Intervention – Enablement
Zhao (216)	China	High	2427		6 to 17			Sleep	Self-report; Parent/proxy report	Sleep duration increased during the week compared to pre-pandemic by about 1 hour, weekend sleep stayed the same.	Sleep increased	Policy – Service Provision

Supplemental Table 2a. Content analysis of future directions based on extracted data – INTERVENTIONS (per the Behaviour Change Wheel, Michie et al., 2011).

Intervention Categories	Future Directions
Education (73.1%, 133 studies)	● Provide information to parents on strategies to promote healthy movement behaviours.<br>● Better understand the relationship between lack of outdoor time and poorer eyesight outcomes in children.<br>● Evaluate the impact of stress and anxiety levels of parents on their children is necessary.<br>● Expand knowledge of the long-term consequences that lockdown measures (low PA, high ST) could have on children's health behaviours.<br>● School nurses and other healthcare personnel are encouraged to educate children and their parents about the importance of getting sufficient sleep and factors that contribute to inadequate sleep among children during the COVID-19 pandemic.<br>● Better understand the mechanisms behind the impact of screens on children’s PA may help to create targeted parental control plans.<br>● Evaluate reasons for changes in PA and ST at home over the subsequent COVID-19 restrictions, including the easing of restrictions.<br>● Better understand whether changes in screen use due to COVID-19 are attenuated, levels vary by child, or if increased screen use is the new normal.<br>● Better understand learning preferences (e.g., screen-based or not) and preferred modes of communication in relation to ST and academic and health outcomes + the impact of educational screen use on academic outcomes.<br>● Find strategies to maintain PA during the current pandemic and future public health crises.<br>● Longitudinal research is needed to capture how families are coping and adapting to the evolving pandemic and to understand if and how positive changes in movement behaviours and daily routines can be maintained post-pandemic.<br>● Study the future consequences of obesity in childhood, both in the short term, causing dyslipidemia, orthopedic problems, insulin resistance, among other conditions; and in the long-term increasing adulthood obesity, cardiovascular diseases, raised cancer risk and a range of other health issues.<br>● Design and evaluate interventions on parenting practices around ST.<br>● Better understand the transmission of COVID-19 in recreational facilities<br>● Further examine parenting challenges during the pandemic and how they would impact children's screen use.

	● Examine whether changes in movement behaviour are enduring or naturally improve with the easing of restrictions and whether these changes have enduring effects on either parent or child mental health, and in turn, more distal risk for psycho-cardiological disease.<br>● Confirm whether longer or repeated movement restriction durations would exacerbate the initial negative 24-hour movement behaviour trends observed, especially for children who are less fit when restrictions are first initiated.<br>● Investigating factors of adherence and non-adherence to the 24-hour movement behaviour guidelines and investigate the long-term consequences of the pandemic on children and youth's health.<br>● Better understand longitudinal emotional impact of the pandemic and movement behaviours.<br>● Long-term follow-up is needed to understand if behavioural changes ultimately contribute to myopia progression.<br>● Better understand the need for educational digital devices, recognize new barriers to healthcare access, and further investigate the effect of the pandemic on physical, psychological, and social well-being of these children, especially in the setting of new variants and high transmissibility which can impact PA, school closure, and transport access.<br>● Further research is needed to unpack what type of support can be put in place to ensure children receive enough activity if physical distancing and health measures remain in place, or in the case of additional waves of this, or another, pandemic. In addition, greater efforts are needed to ensure children's voices are heard regarding the pandemic's impacts on their activity levels and social connectedness.<br>● Our study highlights the need for long-term monitoring of these indices and the development of COVID-19 mitigation measures that carefully incorporate effectiveness and societal impact.<br>● Children-focused interventions should be explored to reduce high ST and tested through an intervention study.<br>● It would be important to assess these same children in the future to ascertain the continuity of their behaviour.<br>● Future research should investigate the association amongst parental stress, children's ST and children's mental health and the lived experience of children as the pandemic continues. Disadvantages and benefits of media and social ST should be explored in relation to mental health outcomes in parents and children during the ongoing COVID-19 pandemic.<br>● Most studies among healthy or representative samples report a weak relationship between PA and ST or other forms of media usage among children and adolescents and speak to the fact that issues among both behaviours need to be addressed separately.<br>● Further research is required to understand the long-term impact of the COVID-19 pandemic on children's eating habits and physical activities. This will help with the implementation of more effective health interventions.<br>● Further research is required on how COVID-19 restrictions may have differentially impacted children from different social groups, including: race and ethnicity, SES and rural/urban.<br>● Further research is required to understand both the immediate and long-term outcomes of COVID-19 restrictions on children.<br>● Further research is required to understand and identify children and young people who are most at risk of engaging in unhealthy movement behaviours during COVID-19 restrictions.<br>● Further research is required to confirm whether children's movement behaviours are maintained over time when social restrictions are removed.<br>● Further research is required to determine how COVID-19 related barriers and facilitators impacted movement behaviours among children.<br>● Further research is required to understand more about parental stress during the pandemic as this would directly impact children's behaviours.<br>● Further research is required to understand how movement restriction, in particular increased time spent at home, impacts children's play behaviours.<br>● Further research is required to understand children's perceptions of safety when using parks during COVID-19.<br>● Further research is required to understand the role of income on children's ST.<br>● Further research on sleep disorders in children during the pandemic is required.<br>● Include more self-reports from the children, comparing their perceptions to their parents.

	● Further study with larger sample size and objective measurements.<br>● Given that disrupted and insufficient sleep has been linked to immune system dysfunction, our findings also have potential implications for resilience to infection in young children during the COVID-19 pandemic. Future studies should further explore deficient sleep as a risk factor for coronavirus infection.<br>● More studies should be conducted to enhance the quality of online or digital-related exercise programs for children with disabilities and to explore the statistical effectiveness of the programs, which will eventually enable children with disabilities to enjoy the benefits of exercise.<br>● Future research should investigate approaches to support educators and ECEC centers to (1) further develop their understanding and knowledge of PA, physical literacy in the context of the early childhood education curriculum, (2) further explore knowledge and implementation strategies to increase the professional practice uptake of healthy eating evidence-based best practice, and (3) increase their use of evaluation tools to measure and track progress.<br>● Longitudinal studies are needed to examine causality including both pre-, during, and post-COVID-19 pandemic data.<br>● Further understanding of parent's mental health, living situations and family compositions.<br>● Future examination of changes in PA before to after the pandemic with a larger sample size.<br>● Further investigation into family behaviours and child PA.<br>● Further understanding of the role of sleep might play in perpetuating any persistent pandemic-related issues.<br>● Need for ongoing mental health monitoring and support for both children and parents. Longitudinal data are required as pandemic waves continue.<br>● Guiding children with their use of electronic devices and helping them eat healthily are important during the COVID-19 pandemic.<br>● Understanding associations between maternal and infant sleep could have shed light on the mechanistic processes underlying the effects of home confinement on sleep of infants and their mothers, and future studies may wish to investigate these links.<br>● Further research on the causal association of PA and dietary habits with overweight and obesity among schoolchildren during the COVID-19 lockdown.<br>● Delivery of effective interventions to reduce ST needs to be developed and enforced to maintain sleep hygiene, and finally overall health.<br>● Further studies are needed to better understand relationships between home space and use of public spaces or outdoors.<br>● Further understanding of lock down measures on infant sleep and underlying mechanisms.
Persuasion (0.6%, 1 study)	● There is a need to find ways to make ST less attractive than PA.
Incentivization (0.6%, 1 study)	● Finding ways to incentivize PA peri- and post-COVID-19 is an important part of the recovery process.
Coercion (0%, 0 studies)	
Training (30.2%, 55 studies)	● Training programs for parents and those who work with children to identify changes in their behaviours from observations in their daily life that might warrant follow-up with a health professional.<br>● Provide information to parents on strategies to promote healthy movement behaviours.<br>● Primary healthcare providers and mental health nurses should increase attention to children's mood and intervene to protect and improve children's mental health during and immediately after the pandemic.<br>● Online training is an effective way to promote PA in the pandemic to children with obesity and authors suggest organizing more active online programs for children.<br>● Specific training programs for children with sleep disorders and their parents should be established.<br>● Health-related awareness programs for students.

	● Enhanced PREVENT yielded promising results, providing a model for family-centered implementation strategies, including telehealth delivery and intervention choice to enhance participation (Family-Based Telehealth Initiative to Improve Nutrition and PA for Children with Obesity).<br>● Support parents with tools to monitor their child's screen use including ways to set rules and expectations, more educational material on PA and family-based activities.<br>● Support parents to encourage children's movement behaviours during times of increased stress and exhaustion such as during COVID-19.<br>● Raise the awareness of parents around extreme ST and digital gaming of children and support them in taking precautions.<br>● Develop resources and strategies to better support children with ST during and after the pandemic.<br>● Develop strategies for at-home learning since ST outside of school hours is also increased.<br>● Encourage parents to engage in joint media use to encourage spending time with their kids than using digital games to distract their kids.<br>● Preventive and educational initiatives may be necessary to mitigate the impact on children's PA in future periods of social distancing.<br>● Infants and caregivers staying at home should be provided special support during the pandemic.<br>● Incorporate flexibility training exercises and interventions to reduce sitting time during online education. This will further strengthen the physical flexibility of students.<br>● Provide effective strategies to promote resilience in the face of disruption during the pandemic.<br>● Families should consider their child's age when exposing them to cell phones, increase development of active ways to engage children with smartphones.<br>● To promote sleep health, a regular schedule for wake time, outside activities and bedtime are recommended. Moreover, electronic devices should be turned off at least 1 h before going to bed, because excessive ST and exposure to blue light before bedtime are associated with poor sleep.
Restriction (1.1%, 2 studies)	● Recognition of the lockdown phenomenon is the first step in taking preventive measures.<br>● Parenting practices that include using more restrictive mediation strategies regulate children's ST. This could be an effective intervention tool used from preschool years.
Environmental Restructuring (14.3%, 26 studies)	● Policies should be made to look at promoting PA and good sleeping habits, and explore the educational opportunities, social media and social support which can be beneficial to the children's health and grooming.<br>● Use of telehealth delivery can support the uptake of interventions during periods of lockdown.<br>● There is thus a critical need to reimagine multisector approaches to providing equitable opportunities for PA, including sports and outdoor recreation, for all children.<br>● The COVID-19 crisis highlights that school fulfills not only an educational mission of knowledge acquisition, but it also satisfies the socialization needs of young people.<br>● Test the determined associations in fathers, as this study sample consisted only of mothers.
Modeling (0%, 0 studies)	
Enablement (17.0%; 31 studies)	● School administrators should prioritize recess as a scheduled opportunity for movement to promote healthy social and physical behaviours.<br>● Enabling interactive learning in schools through videoconferencing might influence the motivation and success of homeschooling.<br>● Tailored distance learning must be established, and children and their parents must be supported according to their individual needs.<br>● School, sport and government authorities need to set up effective strategies promoting school children's PA both during and after COVID-19.<br>● Increase parental support mechanisms to integrate PA in online learning.<br>● Support children to have physically active virtual peer communications and connections.<br>● Increase children and young people's PA opportunities.

	• Increase parental social support in relation to children's movement behaviours.<br>• Efforts are needed to address declines in MVPA, (e.g., providing at-home family PA opportunities) particularly among middle schoolers, as PA patterns established in childhood are likely to continue into adulthood.

Table 2b. Content analysis of future directions based on extracted data – POLICIES (per the Behaviour Change Wheel, Michie et al., 2011).

Policy Categories	Future Directions
Communication/Marketing (14.3%; 26 studies)	• Mental health actions need to be considered essential components of the national response to COVID-19.<br>• Leverage social media to promote/discuss the importance of PA and good sleeping habits.<br>• Inform parents they should have children checked by a healthcare professional when exhibiting behavioural and mood changes or sleep problems.<br>• Target families with health messaging to increase household engagement in PA.<br>• Consider preferred modes of communication when designing campaigns to promote healthy movement behaviours.<br>• Conduct awareness programs through stakeholders' engagement in mitigating negative health impacts.<br>• Develop campaigns and raise awareness for all families to support their children in reducing ST and improving sleep, irrespective of socioeconomic status.<br>• Strategies should be developed and implemented by governments, medias, parents, and teachers as part of the pandemic responses for preserving children's health, for the most socially vulnerable children population.<br>• The city officials should consider how parks and other health infrastructure can be used safely and how to communicate this health and safety information to the public appropriately when COVID-19 and other communicable diseases threaten community health.<br>• Efforts must be made to advocate for an active lifestyle.
Guidelines (40.1%; 73 studies)	• Recommendations re: proper eye-protection measures (particularly in instances of limited outdoor time) are needed.<br>• Support and guidelines are needed for parents and children for balancing ST with family interaction time and PA time.<br>• National guidelines on PA promotion are required to ensure a whole-system approach that involves schools, the home community, and family to help the entire society move in the same direction.<br>• Mental health actions need to be considered essential components of the national response to COVID-19.<br>• Identify protocols that improve children's safety to facilitate children's return to PA programming.

	● Pediatricians should pay attention and, during consultations, inquire how the patient and their family are adapting to life in isolation conditions and what they are feeling. This will allow us to warn about psychosocial risk situations.<br>● These results highlight the need for specific guidelines for promoting healthy digital behaviour in children and preventing these lifestyle changes from becoming permanent once the new normal is achieved.<br>● Understand and identify effective strategies and recommendations that support those most at risk of unhealthy movement behaviours during COVID-19 restrictions.<br>● Provide guidelines to limit screen exposure and to maintain regular sleeping patterns among school-aged children and adolescents.<br>● Need to determine the long-term consequences of public health policies in children and young adults, especially in minority populations, and the importance of developing targeted activity interventions to mitigate potential downstream health consequences.<br>● Given the severe acute changes in response to confinement we observed for young children, it is of utmost importance to raise public awareness and provide timely psychosocial interventions in comparable situations. To this end, public health officials, infectious diseases physicians, psychiatrists and psychologists need to provide sleep-specific guidance to support those families at risk of adverse psychological, social and developmental consequences of confinement. We strongly recommend tracking the well-being of adults as well as young children.<br>● Public health officials and elected leaders should provide clear recommendations for children and families to enable participation in PA outdoors and invest in safe outdoor opportunities (with limits such as physical distancing) to promote healthy movement behaviours in children during the COVID-19 pandemic.<br>● As the global pandemic continues, new guidelines that supplement existing guidelines for children's ST should be developed, and it is expected that policies and plans to support the mental health of vulnerable populations will be prepared. In addition, it would be helpful to provide psychological support, such as counseling services, to alleviate distress and guilt for parents experiencing high levels of depression and stress.<br>● Recommendations and guidelines which detail how to co-exist with digital devises in a healthy and safe way.
Fiscal (0.6%; 1 study)	● Considering multiple and repeat lockdowns and the negative changes in lifestyles observed in children, investments in health promotion are critical to avoid a cascade of negative health consequences in the decades ahead.
Regulation (3.3%; 6 study)	● Policymakers should imperatively consider the balance of disease prevention and promotion of a healthy lifestyle when (re-)activating restrictive measures.<br>● Policies should be made to look at promoting PA and good sleeping habits, and explore the educational opportunities, social media and social support which can be beneficial to the children's health and grooming.<br>● School closures and contact prohibitions should be weighed against negative effects on wellbeing, coping with homeschooling, and leisure behaviour, and be delayed as long as possible.<br>● Government officials and policy makers may want to consider the deleterious lifestyle effects of the lockdown on youths with obesity when making decisions regarding when and how to loosen restrictions.<br>● Policymakers should imperatively consider the balance of disease prevention and promotion of a healthy lifestyle when (re-)activating restrictive measures.<br>● Government officials and policy makers may want to consider the deleterious lifestyle effects of the lockdown on youths with obesity when making decisions regarding when and how to loosen restrictions.
Legislation (0%; 0 studies)	

Environmental/Social Planning (33.5%; 61 studies)	● Support and guidelines are needed for parents and children for balancing ST with family interaction time and PA time.<br>● Increased collaboration among various sectors of children and youth advocates is needed to create comprehensive opportunities for children to engage in PA.<br>● Community stakeholders and policymakers should advocate for safe, equitable access to outdoor spaces by investing funds into maintaining and renovating schoolyards, playgrounds, and public parks.<br>● Create targeted parental control plans for children's ST and PA, which could result in long-term effects such as reducing the risk of obesity in the future.<br>● Strategies to increase activity during confinement must be targeted to each child's specific age group, and conjoint family strategies should be promoted.<br>● Public health strategies for the development and implementation of interventions to promote safe PA and reduce ST in case further lockdowns occur.<br>● Community stakeholders and policymakers should advocate for safe, equitable access to outdoor spaces by investing funds into maintaining and renovating schoolyards, playgrounds, and public parks.<br>● Lifestyle is an important predictor of children's and adolescents' well-being, and future public health strategies aiming to promote well-being should target sufficient time for PA and sleep as well as reduce ST.<br>● Health promotion strategies to help parents facilitate healthy behaviours at home such as having pediatricians provide education and screen about health behaviours.<br>● Policymakers and educators should promote health education during the pandemic including raising awareness on importance of establishing sleep habits.<br>● Policy level change to increase access to indoor and outdoor spaces.<br>● Inform urban planning policy to increase outdoor activity space especially in dense communities (walking, biking, sports facility).<br>● Secure or redirect funds to implement new ways for children to engage in movement behaviours safely during the pandemic, recovery, and post-pandemic phases.
Service Provision (42.9%; 78 studies)	● Particularly, for children and adolescents who are living in lower-income and middle-income countries, efforts to respond to these challenges such as adoption of digital health intervention alongside conventional therapies are recommended.<br>● Regardless of school closures, the range of support services for children and families in need must be expanded and simplified to prevent or counteract the development of serious mental disorders.<br>● Integrating follow-up from observations of children who need to access support services.<br>● Special attention should be paid to groups vulnerable to MVPA reduction as children who have chronic diseases and/or overweight/obesity as well as non-participation in sports.<br>● Online training is an effective way to promote PA in the pandemic to children with obesity and authors suggest organizing more active online programs for children.<br>● Policymakers should address efforts to support and reinforce the relationship within and between families and community services, particularly pediatricians and schools.<br>● Regardless of school closures, the range of support services for children and families in need must be expanded and simplified to prevent or counteract the development of serious mental disorders.<br>● Policymakers should address efforts to support and reinforce the relationship within and between families and community services, particularly pediatricians and schools.

	● Healthcare professionals should assess sleep anxiety with children.<br>● Safe and accessible healthy lifestyle strategies should be developed and implemented by governments, media, parents, and teachers as part of the pandemic responses for preserving children´s health, for the most socially vulnerable children population.<br>● Implement bespoke programs for families that include psychological support to improve pre-school children's sleep time and support their psychological well-being.<br>● Implement PA interventions that target upper secondary school pupils, in particular girls.<br>● Therapy should be continued as well as the close monitoring of clinical manifestation in children with VKC participating in long-distance learning.<br>● Need to make efforts to protect and support healthy behaviours during a period of lockdown in young people.<br>● Public health care during this pandemic must be optimized. However, this requires knowledge not only of the medical and biological sciences, but of all sciences, and then harnessing it to elucidate the human problems that may arise, become aggravated or be perpetuated by the social isolation imposed by the COVID-19 pandemic.<br>● Findings suggest that policy intervention as well as evidence-informed social supports are needed to promote healthful screen use and mental health in children and youth during the pandemic and beyond. These may include resuming in-person learning, reducing electronic-learning time, and encouraging children to reduce screen use. It is also important to ensure that children have access to additional mental health and social support resources and to provide teachers and therapists with training on problematic media use to help.<br>● A program for restoring well-being after the pandemic and supporting pro-development ways of spending free time among adolescents is needed, with consideration for gender differences. Girls, especially in adolescence, need tailored assistance.
